# Characteristics of Adsorption/Desorption Process on Dolomite Adsorbent in the Copper(II) Removal from Aqueous Solutions

**DOI:** 10.3390/ma16134648

**Published:** 2023-06-27

**Authors:** Eleonora Sočo, Andżelika Domoń, Dorota Papciak, Magdalena M. Michel, Dariusz Pająk, Bogumił Cieniek, Mostafa Azizi

**Affiliations:** 1 Department of Inorganic and Analytical Chemistry, Faculty of Chemistry, Rzeszow University of Technology, 35-959 Rzeszow, Poland; eleonora@prz.edu.pl; 2 Department of Water Purification and Protection, Faculty of Civil, Environmental Engineering and Architecture, Rzeszow University of Technology, 35-959 Rzeszow, Poland; dpapciak@prz.edu.pl; 3 Institute of Environmental Engineering, Warsaw University of Life Sciences-SGGW, 02-787 Warsaw, Poland; magdalena_michel@sggw.edu.pl (M.M.M.); mostafa_azizi@sggw.edu.pl (M.A.); 4 Department of Casting and Welding, Faculty of Mechanical Engineering and Aeronautics, Rzeszow University of Technology, 35-959 Rzeszow, Poland; pajak@prz.edu.pl; 5 Institute of Materials Engineering, College of Natural Sciences, University of Rzeszow, Pigonia 1, 35-310 Rzeszow, Poland; bcieniek@ur.edu.pl

**Keywords:** adsorbent, dolomite, copper, heavy metal, isotherm

## Abstract

The removal of hazardous heavy metals that have been released into the environment due to industrial activities has become an important issue in recent years. The presented study concerned the removal of copper(II) ions from aqueous solutions using dolomites. Dolomite is a very attractive adsorbent due to its wide availability, low cost, good adsorption, and environmental compatibility. The paper describes the properties of D-I and D-II dolomites from two different open-cast mines in Poland. The properties of natural adsorbents were determined based on point of zero charges (PZC), elemental analysis of the adsorbent composition, FT-IR, XRD, and SEM spectra analysis. Depending on the initial concentration of the solution used, the adsorption efficiency of copper(II) ions was 58–80% for D-I and 80–97% for D-II. The adsorption mechanism in the case of D-II dolomite was mainly based on ion exchange, while chemisorption dominated the D-I dolomite surface. Considering the possibility of the regeneration and reuse of the adsorbent, dolomite D-II is a better material (the desorption efficiency of copper(II) ions was 58–80%). The adsorption behavior of dolomites has been described using six adsorption isotherms. The best fit was obtained for the Redlich–Peterson, Jovanović, and Langmuir isotherms, indicating that monolayer adsorption occurred. The maximum adsorption capacity for copper(II) was 378 mg/g of D-I and 308 mg/g of D-II.

## 1. Introduction

The rapid development of industrial and economic processes has contributed to the generation of huge amounts of wastewater containing toxic chemicals and heavy metals that pose a threat to aquatic ecosystems and human and animal health [1,2]. Pollution is introduced into natural water from various sources, such as industrial emissions, fossil fuel combustion, inadequate waste disposal, and soil contamination [3,4,5]. Industry related to galvanization processes produces wastewater rich in copper, zinc, silver, gold, and cadmium. These elements are highly soluble in water and easily absorbed by living organisms. Heavy metals can be very toxic and, even at low concentrations, can accumulate in the food chain of animals and humans [6,7,8,9]. Therefore, the study of heavy metal pollution in aquatic ecosystems is essential from the point of view of public health. Heavy metals should be removed as soon as their elevated concentration is detected.

Copper is one of those elements that can pose a real threat to human health. When the permissible concentration is exceeded, copper is very harmful and can cause anemia, kidney and liver damage, gastrointestinal disorders, coma, and even death. This element may also be responsible for the “pink disease” among infants and is carcinogenic to animals. When copper is released into the water, it can eliminate all fish, invertebrates, and marine plants within a radius of many kilometers from the source [10,11,12,13]. Excessive copper retention also causes Wilson’s disease, which causes neurobehavioral abnormalities similar to schizophrenia [14]. The Environmental Protection Agency and World Health Organization have defined the maximum permissible range for copper(II) in potable water as 1.0–2.0 mg/dm^3^ [15].

The presence of toxic metals in sewage entering natural water leads to the need to develop technologies for their removal and recovery [16]. Many separation methods have been developed, including solvent extraction, co-precipitation, membrane filtration, biosorption, ion exchange, and adsorption. These methods are used to remove heavy metal ions from various aqueous solutions with a wide range of concentrations. Although they are considered traditional methods, some cannot meet the new and more stringent requirements and/or are expensive. Therefore, there is a growing need to develop new, innovative, and cost-effective methods of removing heavy metals from wastewater [17,18,19].

Numerous studies have been carried out to develop cheaper and more effective technologies to reduce the amount of wastewater produced and improve the quality of treated wastewater. Adsorption has become a popular method of water purification due to its efficiency (it can be used to remove contaminants in low concentrations) and can be used in both batch and continuous systems. In addition, it is easy to use, generates small amounts of post-process waste, and it is possible to regenerate and reuse adsorbents. Adsorption is one of the most cost-effective treatment technologies, but the adsorbents used can be very expensive. Therefore, there is a need to search for suitable adsorbents that will be both easy to obtain, cheap, and effective in removing heavy metals.

In recent years, many studies have been conducted on the use of natural materials, rock minerals, or agricultural waste as potential adsorbents. The use of highly efficient, environmentally friendly, and inexpensive materials for the adsorptive removal of pollutants from water has always been seen as a new task. In order to remove dyes and heavy metals from aqueous solutions, coal dust [20,21], tea waste [22,23], dolomite [17,22], activated carbon [24,25], silica [26], clays [27], chitosan [28], and zeolites and their derivatives [29,30,31] were tested. Various types of agricultural waste, which can also be transformed into catalysts with adsorption properties, were also tested [2]. Agricultural waste is an attractive source of raw materials that can be used to develop more ecological and economical methods of water treatment. It was confirmed that some of the locally available materials can be used as cheap adsorbents.

Based on the above considerations, the main aim of the work was to assess the effectiveness of copper removal from water solutions on natural adsorbents, which were dolomites (carbonate minerals). The obtained test results made it possible to determine the stability of the bond between copper(II) ions and the adsorbent and the possibility of adsorbent regeneration. Moreover, the extent to which the process of adsorption and desorption of copper(II) ions affects the structure of dolomites was checked. Interpreting the obtained results made it possible to assess the validity of using carbonate minerals (dolomite) for water or wastewater purification. Mineral adsorbents extracted in two mines located in Poland, i.e., dolomite from the Józefka open-cast mine (D-I) and dolomite from the Piskrzyn mine (D-II), were used for the study.

## 2. Materials and Methods

### 2.1. Subject of Study

Mineral adsorbents extracted in two mines located in Poland, i.e., dolomite from the Józefka open-cast mine (D-I) (50°50′18.07″ N; 20°49′6.08″ E) and dolomite from the Piskrzyn open-cast mine (D-II) (50°45′22.66″ N; 21°16′21.48″ E), were used for the study (Table 1). In order to ensure equal testing conditions, both dolomites were sieved through a sieve with a mesh size of 0.3 mm.

### 2.2. Physicochemical Properties of Dolomites

A detailed study was conducted to evaluate the dolomite structure, chemical composition, and properties. The samples of dolomite were subjected to the following set of analyses:

Analysis of dolomite morphology using a Keyence VHX 7000 microscope (Mechelen, Belgium).

Determination of the pH value of water solutions of the adsorbents:

To determine the pH value of water solutions of the adsorbents, 0.5 g of dolomite and 50 cm^3^ of distilled water were weighed in two beakers. The contents of the beakers were mixed and then the pH of the solution was measured.

Determination of the zero point of charge of the dolomites:

Suspension method: A total of 0.5 g of dolomite and 50 cm^3^ of 0.1 M NaNO_3_ were weighed into ten flasks with a capacity of 100 cm^3^. Using HNO_3_ and NaOH solutions, the following pH values were determined, 2; 3; 4; 5; 6; 7; 8; 9; 10, and 11 (pH_0_). The samples were stirred for 8 h using a magnetic stirrer, then the pH of the samples (pH_1_) was measured again and the value of the expression, ΔpH = pH_1_ − pH_0_, was calculated.

Potentiometric method: A total of 0.5 g of dolomite and 50 cm^3^ of distilled water were weighed into 100 cm^3^ flasks, then the samples were titrated with 0.1 M HCl. The point of zero charges was determined at the intersection of the titration curves for the analyzed and the blank samples. Due to the lower value of the starting pH, the blank sample was titrated with a standard solution of 0.01 M HCl.

### 2.3. Batch Adsorption and Desorption Experiment

To characterize the adsorption of copper(II) ions on dolomite, 0.5 g of dolomite was added to 50 mL of copper(II) nitrate(V) trihydrate solution with copper(II) ion concentrations of 3000, 2000, 1000, 500, 250, 100, and 50 mg/dm^3^. The pH of the solutions was adjusted to 7 to obtain the copper(II) ions as the main component and neutral environmental conditions. The mixtures were agitated for 2 h on an Elpin lab shaker (vibration amplitude: 8 mm; vibration rates: 180 rpm) and then the mixtures were filtered into flasks using a filter set. The concentration of copper ions was measured using a FAAS spectrophotometer (wavelength was 324.8 nm).

The deposits remaining on the filters resulted from filtering samples after the adsorption on dolomite were dried and then quantitatively transferred to 100 cm^3^ flasks. A total of 50 cm^3^ of distilled water was added to each flask, and then the resulting solutions were brought to pH = 1 with concentrated nitric acid (V). The contents of the flasks were shaken for 2 h, then the solutions were filtered into the flasks using a filter set, and the copper concentration was determined using a FAAS spectrophotometer.

### 2.4. Chemisorption and Ion Exchange—Determination of the Copper(II) Adsorption Mechanism on Dolomite

The analysis of the chemisorption and ion exchange process allows us to determine the interactions between the adsorbent structure and the adsorbed copper(II) ions, and thus, to determine the scale of the desorption process. To determine the occurrence of chemisorption and ion exchange, the content of copper(II) ions, expressed in volume units [mg/dm^3^], was converted into mass units [mg/g], according to the following formulas:

(1) $$Chemisorption=\frac{{(C}_{ads}-C_{e,des})\cdot V}{m}$$

(2) $$IonExchange=\frac{C_{e,des}\cdot V}{m}$$

where C_ads_—concentration of copper(II) ions adsorbed on the adsorbent, [mg/dm^3^]; C_e,des—_concentration of copper(II) ions in the aqueous solution after the desorption process, [mg/dm^3^]; V—the volume of solutions subjected to desorption, [dm^3^]; and m—the adsorbent mass used for desorption, [g].

### 2.5. Analysis of Changes in the Structural Properties of Dolomites after the Process of Adsorption and Desorption

In order to determine changes in the properties of dolomites after the adsorption and desorption process, the following analyses were performed:

Elemental analysis of the composition of dolomites using the PerkinElmer CHNSO 2400 elemental analyzer.

X-ray diffraction analysis (XRD): The mineral composition of dolomite was assessed using X-ray diffraction (Bruker D8 Advance, Bruker AXS, Karlsruhe, Germany).

SEM microscopic analysis: The morphological and textural observation of the surface was made using a scanning electron microscope (SEM) (TESCAN VEGA 3, Fuveau, France). The SEM was also used with a back-scattered electron detector (BSE) (INCA x-act, Oxford Instruments) to broaden the scope of the element content analysis. 

Fourier transform infrared spectroscopy (FT-IR) analysis: FT-IR spectra of dolomite were obtained with an Alpha spectrometer (Bruker, Billerica, MA, USA).

### 2.6. Determination of Adsorption and Desorption Isotherms

The adsorption properties of the tested dolomites were assessed using six empirical isotherm equations presented in Table 2. The isotherm that most accurately describes the studied phenomenon was selected based on the data calculated by the Origin 7.5 program.

The determination coefficient (R^2^) and the reduced chi-square test (error analysis) were used as the criterion for selecting the appropriate equation, allowing for determining to what extent the determined curve matches the specified experimental data. The equation describes the reduced chi-square test:

(3) $$\chi^{2}/DoF=\frac{1}{d}\sum \frac{{\left(C_{e}-C_{e,M}\right)}^{2}}{C_{e,M}}$$

where d—number of degrees of freedom; χ^2^⁄DoF—an indicator describing the error of matching the experimental data of a model; C_e_—concentration value of the test substance in the liquid phase, [mg/dm^3^]; and C_e,M_—the value of adsorbed substance concentration in relation to the model curve, [mg/dm^3^].

## 3. Results and Discussion

### 3.1. Characteristics of Adsorbents’ Properties

**Figure 1:**
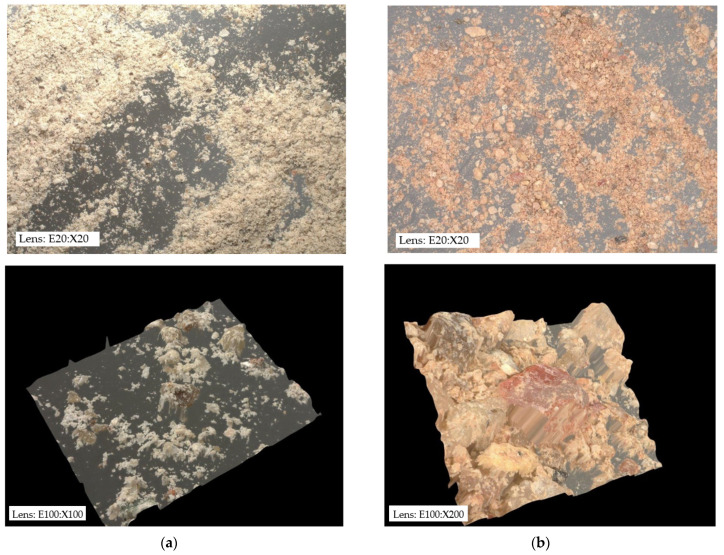
Structure of the analyzed adsorbents: (**a**) D-I, (**b**) D-II.

Dolomite with the chemical formula CaMg(CO_3_)_2_ belongs to the group of carbonate minerals. This mineral is in the form of trigonal, rhombohedral crystals, which can be fine or microcrystalline. Dolomites can occur in various color ranges from white, grey, light brown, red, and green to black. The dolomite from the Józefka open-pit mine (D-I) is gray, while the dolomite from the Piskrzyn open-pit mine (D-II) is brown (Figure 1).

**Figure 2:**
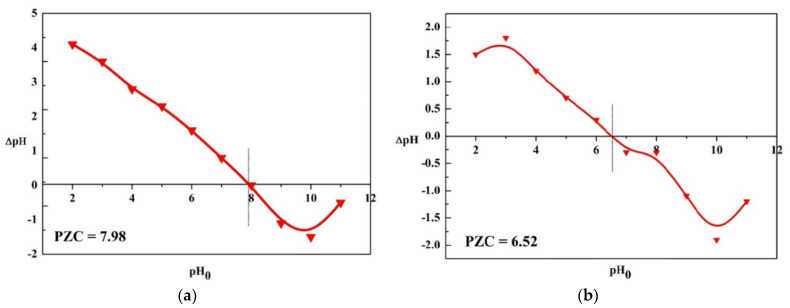
PZC value determined by the suspension method: (**a**) D-I, (**b**) D-II.

**Figure 3:**
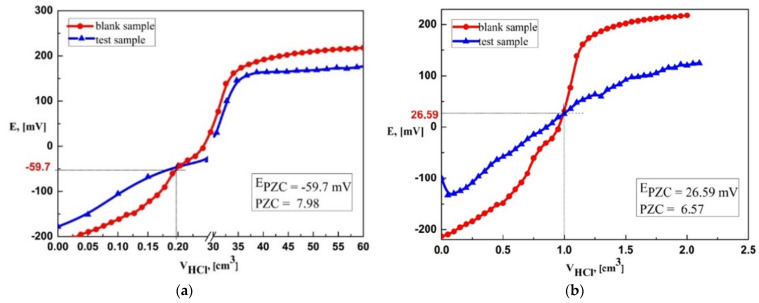
PZC value determined by the potentiometric method: (**a**) D-I, (**b**) D-II.

The average pH value of the aqueous solutions of the analyzed dolomites was 7.85 (D-I) and 7.06 (D-II) (Table 3). The point of zero charges (PZC) is one of the most important parameters to describe the surface properties of solids in aqueous solutions. PZC is the pH value at which a suspension of a solid in water has an electrical charge of zero (the sum of positive and negative surface charges is zero). If the pH of aqueous solutions of dolomites is above PZC, it means that its surface is negatively charged as a result of the ionization of acidic groups (mainly carboxylic, phenolic, and hydroxyl). This phenomenon causes an electrostatic attraction between the metal ions and the negative sites on the adsorbent surface, increasing the adsorption. In addition, when the pH increases, there are fewer hydrogen ions in the solution, the competition for metal ions decreases and adsorption occurs with greater efficiency. However, when the pH is lower than PZC, the surface is positively charged and can exchange anions [38]. The PZC determined by the suspension method was 7.98 (D-I) and 6.52 (D-II) (Figure 2). Similar PZC values were obtained using the potentiometric method (PZC is at the intersection of the titration curves of the samples tested with the blank sample). PZC for dolomite II was 6.57 (potential E = 26.59 mV), and for dolomite, I was 7.98 (potential E = −59.7 mV) (Figure 3). The pH value at which the PZC value is found depends on the nature of the surface of the solid. The obtained values show that the surface of D-II is negatively charged under the experimental conditions of pH 7 and, therefore, will show a greater ability to adsorb metal ions.

### 3.2. Analysis of the Adsorption and Desorption Process on Dolomites

**Figure 4:**
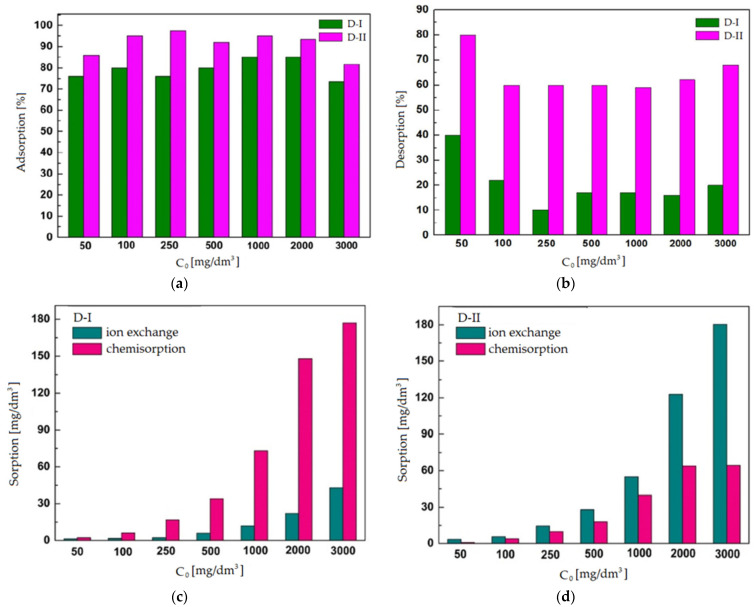
Analysis of the adsorption (**a**) and desorption process (**b**) on D-I and D-II dolomites and the relationship between chemisorption and ion exchange values of copper(II) ions for D-I (**c**) and D-II (**d**).

**Figure 5:**
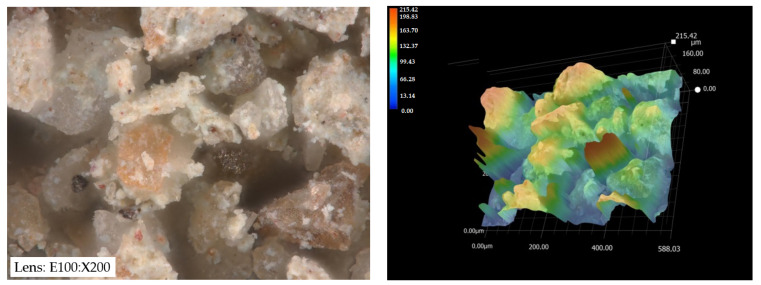
Structure of D-I dolomite after the adsorption process.

The test results showed that dolomite D-II has a greater adsorption capacity toward copper(II) ions (Figure 4). The adsorption efficiency for D-II ranged from 80 to 97%, thus indicating a more developed surface of this adsorbent or a greater number of active sites on its surface. The analysis of the desorption process showed that adsorbed cations could be more easily removed from the surface of D-II dolomite (Figure 4). A higher percentage of desorption (58–80%) indicates that copper(II) ions could be bound to the D-II surface by van der Waals forces (these bonds are more easily broken) or ion exchange took place. On the other hand, dolomite D-I obtained a lower percentage of desorption (10–40%), which proves the predominance of chemical bonds between the dolomite structure and copper(II) ions (chemical bonds require much more energy to break them). The determination of chemisorption and ion exchange rates confirmed the above considerations. In the case of D-I, there are chemical interactions between the adsorbent and the adsorbate. In turn, dolomite D-II was characterized by a greater capacity for ion exchange (Figure 5). Conducting a comprehensive analysis covering both the adsorption and desorption process is of key importance for the practical applications of dolomite because we receive information both on the effectiveness of sorbents in removing copper(II) ions, as well as on the possibility of their reuse.

**Figure 6:**
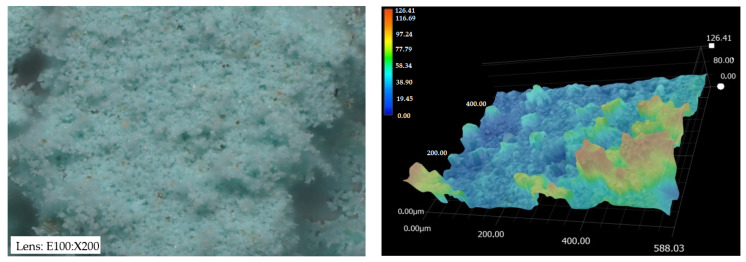
Structure of D-II dolomite after the adsorption process.

After the adsorption process, changes in the structure and composition of the analyzed dolomite samples were observed. Changes in the physicochemical properties of the dolomite are the result of the impact of impurities on the sorbent. As a result of the adsorbed copper(II) ions, the color of dolomite D-II changed from gray to blue, and the spatial structure of this sorbent became more even (elevations in the 0–60 µm range prevail) (Figure 5). The color change may be the result of the formation of copper(II) complexes on the surface of the dolomite or a change in the crystal structure of the sorbent. In the case of dolomite D-I, the color did not change and the structure was characterized by greater unevenness (0–215 µm) (Figure 6). The SEM analysis of the dolomites after the adsorption process confirmed the better adsorption efficiency using D-II dolomite. The literature data indicate that dolomite, despite having a relatively small specific surface area (SBET = 1.52 m^2^/g), shows excellent adsorption properties [20,39].

### 3.3. Elemental Analysis of the Composition of the Dolomites

The analyzed adsorbents in their theoretical composition should contain only CaCO_3_ and MgCO_3_. The elemental analysis of the initial D-I dolomite sample showed the highest content of hydrogen (52%) and sulfur (26%). In contrast, in the case of D-II dolomite, it was hydrogen (11%) and nitrogen (7%) (Table 4). The presence of other elements in the structure of natural dolomite proves the presence of impurities on the surface of the adsorbent caused by the repeated processing, extraction, and good adsorption properties. After the adsorption process, an increase in the nitrogen content in the structure of the analyzed adsorbents was noted (for D-I by 41%, for D-II by 5%). The increase in the nitrogen content proves the adsorption on the surface of the dolomites of nitrogen from the copper(II) nitrate(V) trihydrate used for the tests (Table 4). After the desorption process, the content of elements decreases, and the largest decrease occurred for nitrogen (D-I: from 46% to 11%; D-II: from 12% to 2%) and sulfur (D-I: from 36% to 0.5%; D-II: from 8% to 1%). As a result of desorption, the surface of the adsorbent was cleaned, and the adsorbed ions returned to the water phase.

### 3.4. SEM-SE Analysis of the Dolomites

**Figure 7:**
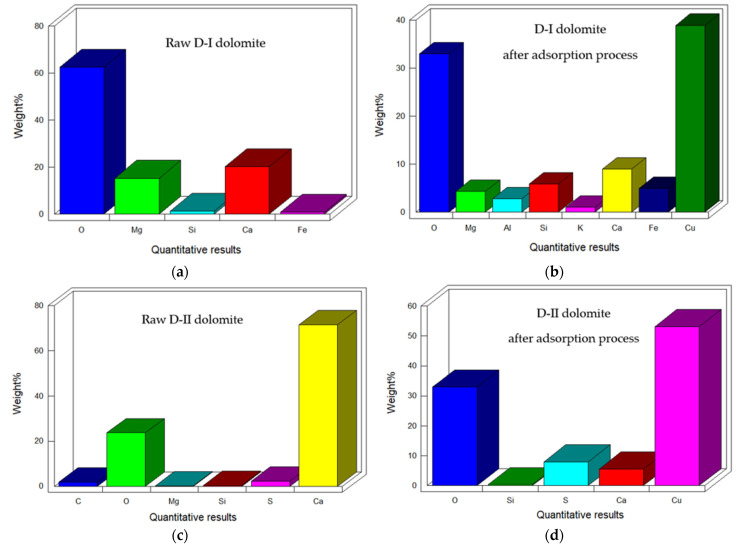
Percentage of elements in raw D-I and D-II dolomite (**a**,**c**), and dolomite after the adsorption process (**b**,**d**).

Based on microscopic dolomite images, BSE spectra were made, thanks to which the elemental compositions of the adsorbents (other than nitrogen, carbon, sulfur, and hydrogen) were determined. The results indicate that the D-I dolomite contained oxygen, calcium, magnesium, and trace amounts of silicon and iron in its composition (Figure 7). However, in the composition of the D-II dolomite, the presence of calcium, oxygen, and a small content of sulfur, carbon, magnesium, and silicon was found. The presence of sulfur or iron may indicate a contaminated dolomite surface (Figure 7). Dolomite as a pure variety rarely occurs in nature. Dolomite may often contain certain amounts of FeO (it then assumes a color ranging from yellow to brown) or MgO (pink shades) [40,41].

**Figure 8:**
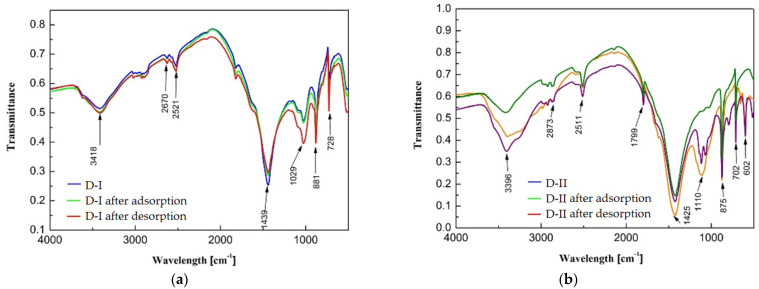
FT-IR spectrum of the initial dolomite and after the adsorption and desorption process: (**a**) D-I, (**b**) D-II.

D-I dolomite, after adsorption, contained the largest amount of copper (38%), oxygen (34%), and calcium (20%). In the D-I structure, an increase in the content of iron (by 5%) and silicon (by 7%) was found, and a small content of potassium and aluminum was noted (Figure 8). After the adsorption process, the structure of D-II dolomite was characterized by the highest content of copper (55%) and oxygen (33%). There was also an increase in sulfur and silicon content and a decrease in calcium content. Magnesium and carbon were not detected during the analysis (Figure 7). The high content of copper in D-I and D-II dolomite confirms that the adsorption process occurred during the experiment. In addition, in the case of D-II, a significant decrease in the calcium content (from 75% to 5%) indicates the removal of copper(II) ions by ion exchange.

### 3.5. Interpretation of FT-IR and XRD Spectra of Dolomites

The FT-IR spectra indicate that the adsorption process is followed by a decrease in the transmittance value, which indicates the adsorption of copper(II) ions on the surface of the dolomites. For D-II dolomite, the splitting of the band at 1110 cm^−1^ into two bands: 1117 and 1068 cm^−1^, can be observed, which confirms the better adsorption efficiency of copper(II) ions on this adsorbent (Figure 8). After the desorption process, the spectrum obtained for D-I dolomite has a lower transmittance value, which indicates no return to the original structure (small degree of desorption). The spectrum of D-II dolomite after the desorption process, compared to the initial spectrum, confirms that in the case of this adsorbent, copper(II) ions interact with the surface based on ion exchange (bands: 1117, 1068, 602 cm^−1^ disappear in the spectrum, and transmittance is also increased). The structure of D-II dolomite after the desorption process differs from the initial structure; therefore, the ions contaminating the original surface of the dolomite could also be desorbed (Figure 8). The analysis of the output spectra of the adsorbents allows for the determining of the presence of characteristic bands occurring in the case of dolomites (calcium and magnesium carbonates). The characteristics of the type and intensity of the most important vibrations obtained on the FT-IR spectra of the analyzed dolomites are presented in Table 5. The FT-IR technique enables the identification of important functional groups that are capable of adsorbing metal ions.

**Figure 9:**
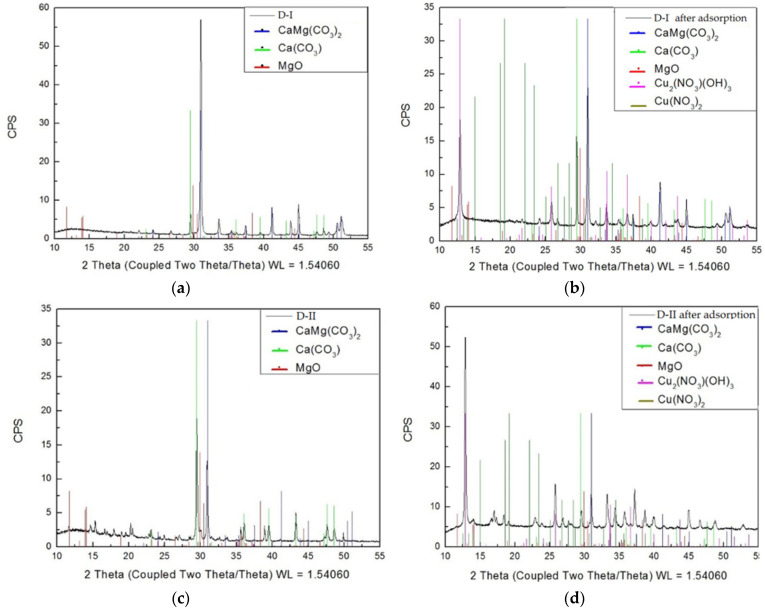
XRD spectra for D-I and D-II dolomite before (**a**,**c**) and after the adsorption process (**b**,**d**).

Analysis of the phase composition of dolomite samples showed that the main phases are: CaMg(CO_3_)_2_, CaCO_3_, and MgO (weaker reflections were obtained for the D-II dolomite) (Figure 9). In the case of the D-I diatomite, a high content of CaCO_3_ (approximately 50%) was noted, while in the composition of the D-II diatomite, the content of this compound was only below 5%. The analysis of the D-I dolomite sample after adsorption showed a decrease in the reflex intensity at 31 degrees 2theta (CaMg(CO_3_)_2_) and an increase in the reflex intensity at 29 degrees 2theta (CaCO_3_). In addition, reflections of approximately 12 degrees 2theta (Cu_2_(NO_3_)(OH)_3_) and faint reflections from Cu(NO_3_)_2_ appeared. The analysis of dolomite D-II showed the appearance of a strong reflection from Cu_2_(NO_3_)(OH)_3_ (approximately 12 2theta), which indicates that the sample consists mainly of Cu (over 50%). Adsorption caused a quantitative decrease of the CaMg(CO_3_)_2_ phase in both analyzed dolomite samples by approximately 40% and contributed to the appearance of the Cu(NO_3_)_2_ and Cu_2_(NO_3_)(OH)_3_ phases. The D-II dolomite showed a higher share of Cu phases (over 50%) than the D-I dolomite (below 20%) (Figure 9).

### 3.6. Adsorption and Desorption Isotherms

**Figure 10:**
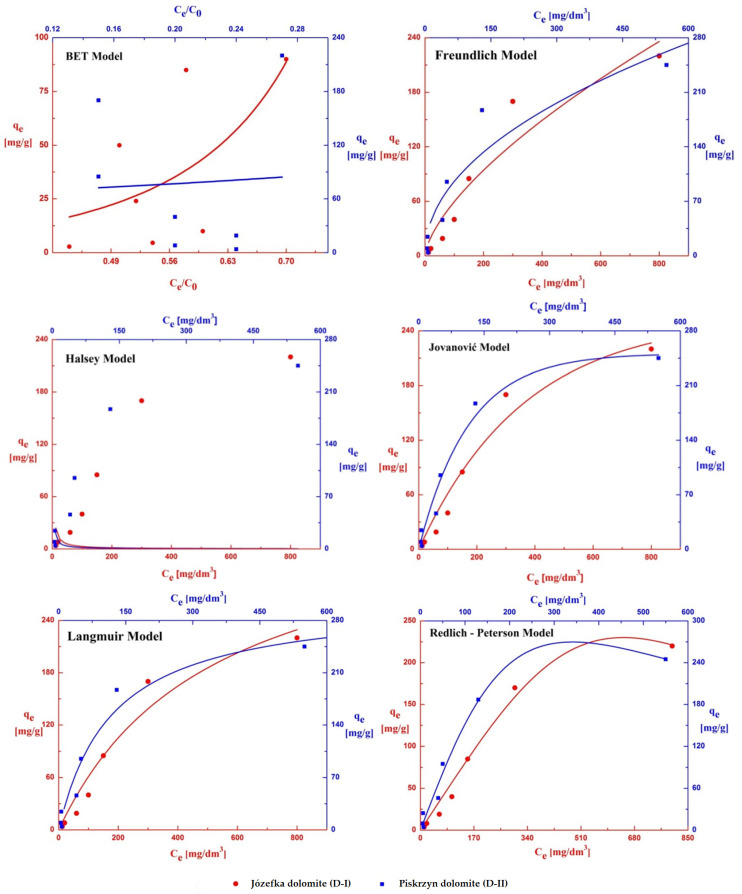
Adsorption isotherms of copper(II) on the D-I and D-II dolomites.

**Figure 11:**
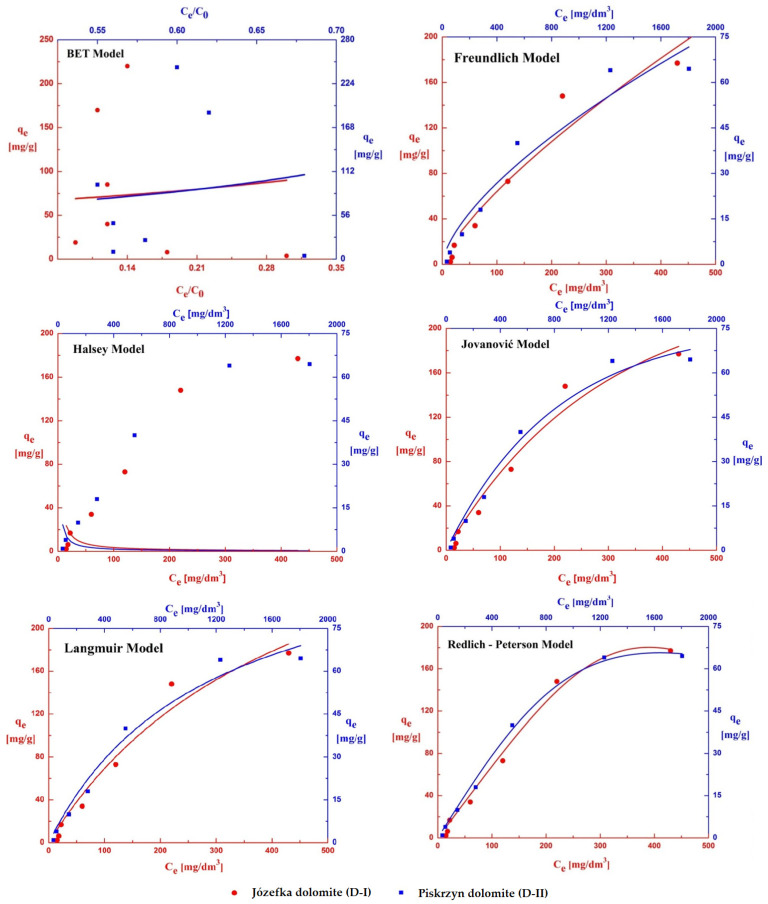
Desorption isotherms of copper(II) on the D-I and D-II dolomites.

The study of adsorption and desorption isotherms allows for a better understanding of the mechanism of these processes and the interactions between the adsorbate and the adsorbent. The matches of six isotherms, i.e., the BET, Freundlich, Halsey, Jovanovic, Langmuir, and Redlich–Peterson isotherms were examined for the obtained experimental data (Figure 10 and Figure 11). Thanks to the mathematical models used, it was possible to calculate, e.g., isotherm constants, the coefficients of determination (R^2^) values, and the value of the reduced chi-square test (χ^2^/DoF), the main isotherm selection criteria. Table 6 and Table 7 show that the Redlich–Peterson isotherm best fits the experimental data. In this case, the coefficient of determination (R^2^) reached the highest values for the adsorption and desorption process (0.985 and 0.999 for D-I; 0.984 and 0.966 for D-II). On the other hand, for χ^2^/DoF, the smallest values ranging from 4.3–211 were obtained, which proves a well-fitted model (Table 6 and Table 7). Suitable matches were also shown by the following isotherms: Jovanovic, Freundlich, and Langmuir; therefore, it can be concluded that a monolayer of adsorbate was formed on the surfaces of the analyzed adsorbents (monolayer adsorption theory). The remaining BET and Halsey isotherms assume the occurrence of multilayer adsorption—in the discussed case, no fit of the experimental curves was found.

The maximum values of the adsorption capacity (q_max_) found in the isotherm equations, BET, Jovanovic, and Langmuir, were also determined. The highest q_max_ values for the adsorption process were obtained for the Langmuir isotherm, which was 378 mg/g for D-I, and 308 mg/g for D-II. The maximum value of occupying the surface of the adsorbents with copper(II) ions showed that dolomite D-I had a higher adsorption capacity. In addition, the heterogeneity parameter (n) and its reciprocal (1/n) were determined, which led to the determination of the ideality of the surface. In the adsorption and desorption process carried out on D-I dolomite, the value of the heterogeneity coefficient in the Freundlich equation ranged from 1.5 to 1.3 (1/n = 0.67 ÷ 0.77). The obtained values show that the surface of the adsorbent is close to homogeneous. In the case of dolomite D-II, the value of the heterogeneity coefficient is in the range from 2.1 to 1.5 (1/n = 0.48 ÷ 0.67), which means that there is incomplete homogeneity on its surface.

Studying both the adsorption and desorption processes is important for a fuller understanding of the processes taking place and for assessing the effectiveness of the sorbent. This allows for the assessment of the sorbent’s ability to capture copper(II) ions from the environment and for the possible regeneration and reuse of the sorbent.

Adsorption of heavy metals from aqueous solutions on natural materials, including dolomites, is a promising technique. This method has many advantages, including low costs, the possibility of managing redundant waste, and the possibility of the biodegradation of the biomass used. In addition, the essential advantages of the adsorption process include the possibility of multiple uses of the adsorbent and the recovery of metals by desorption. The adsorption capacity of natural adsorbents depends on many factors, such as the type of adsorbent, its structure, particle size, pH of the solution, temperature, or contact time. Numerous studies have been conducted on using natural adsorbents or adsorbents made from waste materials, and exemplary maximum adsorption capacity values are presented in Table 8.

## 4. Conclusions

The removal of heavy metal ions from wastewater is crucial from the point of view of environmental protection. The structural properties of the tested dolomites and their adsorption capacity indicate that they can be used as cheap adsorbents to remove copper(II) ions from aqueous solutions (e.g., for the pre-treatment of industrial water).

The adsorption efficiency of copper(II) ions from aqueous solutions on dolomites ranged from 58 to 97%, depending on the analyzed adsorbent and the concentration of the initial solution. The D-II dolomite was characterized by higher adsorption capacity (the efficiency of removed ions was 80–97%), proving a more developed surface of the adsorbent or a greater number of active sites on its surface than the D-I dolomite. The surface of the D-II dolomite is negatively charged and, therefore, can adsorb copper(II) ions.

The adsorption mechanism in the case of D-II dolomite was mainly based on ion exchange. In contrast, the D-I dolomite retained copper(II) ions on its surface using chemical bonds that were more difficult to break. Considering the possibility of regeneration and the reuse of the adsorbent, dolomite D-I is a better material. Equilibrium analysis of the adsorption process was best described using Redlich–Peterson (R = 0.985), Jovanovic (R = 0.962), and Langmuir (R = 0.955) isotherms. On this basis, it was found that the conducted adsorption process was monolayer adsorption. According to the adsorption isotherm equation, it was found that the maximum adsorption capacity of dolomite from the Józefka open-cast mine (D-I) was 378 mg/g and of dolomite from the Piskrzyn open-cast mine (D-II) was 308 mg/g.

## Figures and Tables

**Table 1:** Properties of the analyzed dolomites (manufacturer’s data).

Properties	Dolomite	
	Józefka Open-Cast Mine (D-I)	Piskrzyn Open-Cast Mine (D-II)
Color	gray, white, yellowish, colorless	gray, white
Density (g/cm^3^)	2.8–2.9	2.9–3.0
Gloss	glassy, sometimes pearly	matte
Hardness on the Mosh scale	3.5–4	3–3.5

**Table 2:** Lists of adsorption isotherm models [32,33,34,35,36,37].

Isotherms	Equation	Abbreviations
Brunauer, Emmet, Teller (BET)	$q_{e}=q_{max}\cdot \frac{K_{BET}\cdot \frac{C_{e}}{C_{0}}}{\left(1-\frac{C_{e}}{C_{0}}\right)\cdot \left[1+\left(K_{BET}-1\right)\cdot \frac{C_{e}}{C_{0}}\right]}$	C_o_—the initial concentration of the substance in the solution [mg/dm^3^]. C_e_—the concentration of the adsorbate in the solution at equilibrium [mg/dm^3^]. q_e_—the equilibrium concentration of the adsorbate on the adsorbent surface [mg/g]. q_max_—maximum adsorption capacity [mg/g]. K_BET_—BET adsorption equilibrium constant. K_F_—Freundlich adsorption equilibrium constant [dm^3^/mg]. K_L_—Langmuir adsorption equilibrium constant [dm^3^/mg ]. K_J_—Jovanovich adsorption equilibrium constant [dm^3^/mg ]. K_H_—Halsey adsorption equilibrium constant [dm^3^/mg ]. n—adsorption intensity. a_RP,_ B—Redlich–Peterson constant [dm^3^/mg]. K_RP_—Redlich–Peterson adsorption equilibrium constant [dm^3^/mg].
Freundlich	$lnq_{e}=lnK_{F}+\frac{1}{n}lnC_{e}$	
Langmuir	$q_{e}=q_{max}\cdot \frac{K_{L}\cdot C_{e}}{1+K_{L}\cdot C_{e}}$	
Jovanovic	$q_{e}=q_{max}\cdot (1-\exp\left(-K_{J}\cdot C_{e}\right))$	
Halsey	$q_{e}={\left(\frac{K_{H}}{C_{e}}\right)}^{\frac{1}{n}}$	
Redlich–Peterson	$q_{e}=\frac{K_{RP}\cdot C_{e}}{1+a_{RP}\cdot {\left(C_{e}\right)}^{B}}$	

**Table 3:** pH values of aqueous solutions of the dolomites.

Dolomite	pH Values			Average pH Value
D-I	7.90	7.85	7.80	7.85
D-II	7.06	7.08	7.04	7.06

**Table 4:** Elemental analysis of dolomites (raw dolomite and dolomite after the adsorption/desorption process).

Element	D-I			D-II		
	Raw Dolomite	After Adsorption	After Desorption	Raw Dolomite	After Adsorption	After Desorption
	Percentage of the Element, [%]					
Nitrogen	5	46	11	7	12	2
Carbon	0.4	3	2	1	0.1	0.3
Sulfur	26	36	0.5	2	8	1
Hydrogen	52	14	4	11	12	10
C/N ratio	5	34	9	9	12	2

**Table 5:** Vibration frequencies in the FT-IR spectrum of dolomites.

D-I Dolomite	D-II Dolomite	Intensity	Identification	Wavenumber Range [cm^−1^] [42]
Peak [cm^−1^]				
3418	3396	medium	Stretching bands ν O-H; confirm the presence of water molecules in the dolomite structure	3400–3600
2670 2520	2873 2511 1799	weak	Combination bands characteristic of the dolomites.	approximately 2500 and 1700
1438	1425	strong	Asymmetric stretching bands ν _as_ C-O confirm the presence of compounds such as carbonates.	1400–1500
1029	1110	medium	Stretching asymmetric ν _as_ Ca-O-Ca, Mg.	900–1300
881 728	875 702	medium	Stretching symmetric ν _s_ Ca-O-Ca, Mg.	600–800
-	602	medium	Deformation δ Ca-O-Ca, Mg.	500–600

**Table 6:** The results of matching isotherms to experimental data for the copper(II) adsorption process on dolomites (pH~7).

Isotherms	D-I Dolomite					D-II Dolomite				
BET	R^2^	χ^2^/DoF	K_BET_ [dm^3^/mg]	q_max_ [mg/g]		R^2^	χ^2^/DoF	K_BET_ [dm^3^/mg]	q_max_ [mg/g]	
	0.423	969	0.026	470		<0.1	8802	2.45 × 10^43^	62	
Freundlich	R^2^	χ^2^/DoF	K_F_[dm^3^/mg]	n		R^2^	χ^2^/DoF	K_F_ [dm^3^/mg]	n	
	0.916	739	2.81	1.5		0.897	1100	12.8	2.1	
Halsey	R^2^	χ^2^/DoF	K_H_ [dm^3^/mg]	n		R^2^	χ^2^/DoF	K_H_ [dm^3^/mg]	n	
	<0.1	17149	0.26	9.0 × 10^−4^		<0.1	21,071	0.22	1.7 × 10^−3^	
Jovanovich	R^2^	χ^2^/DoF	K_J_ [dm^3^/mg]	q_max_ [mg/g]		R^2^	χ^2^/DoF	K_J_ [dm^3^/mg]	q_max_ [mg/g]	
	0.962	332	0.003	255		0.975	272	0.009	251	
Langmuir	R^2^	χ^2^/DoF	K_L_ [dm^3^/mg]	q_max_ [mg/g]		R^2^	χ^2^/DoF	K_L_ [dm^3^/mg]	q_max_ [mg/g]	
	0.955	398	0.002	378		0.962	405	0.008	308	
Redlich–Peterson	R^2^	χ^2^/DoF	K_RP_ [dm^3^/mg]	a_RP_ ([dm^3^/mg])^B^	B	R^2^	χ^2^/DoF	K_RP_ [dm^3^/mg]	a_RP_ ([dm^3^/mg])^B^	B
	0.985	166	0.57	2.07 × 10^−8^	0.85	0.984	211	1.69	2.0 × 10^−5^	0.97

**Table 7:** The results of matching isotherms to experimental data for the copper(II) desorption process on dolomites (pH~1).

Isotherms	D-I Dolomite					D-II Dolomite				
BET	R^2^	χ^2^/DoF	K_BET_ [dm^3^/mg]	q_max_, [mg/g]		R^2^	χ^2^/DoF	K_BET_ [dm^3^/mg]	q_max_ [mg/g]	
	<0.1	9446	3.26 × 10^45^	63		<0.1	10,928	9.77 × 10^44^	34.5	
Freundlich	R^2^	χ^2^/DoF	K_F_ [dm^3^/mg]	n		R^2^	χ^2^/DoF	K_F_ [dm^3^/mg]	n	
	0.945	332	2.04	1.3		0.950	45	0.54	1.5	
Halsey	R^2^	χ^2^/DoF	K_H_ [dm^3^/mg]	n		R^2^	χ^2^/DoF	K_H_ [dm^3^/mg]	n	
	<0.1	11760	0.33	9.3 × 10^−4^		<0.1	2035	0.32	1.02 × 10^−3^	
Jovanovich	R^2^	χ^2^/DoF	K_J_ [dm^3^/mg]	q_max_ [mg/g]		R^2^	χ^2^/DoF	K_J_ [dm^3^/mg]	q_max_ [mg/g]	
	0.72	167	0.004	235		0.985	14	0.001	76	
Langmuir	R^2^	χ^2^/DoF	K_L_ [dm^3^/mg]	q_max_ [mg/g]		R^2^	χ^2^/DoF	K_L_ [dm^3^/mg]	q_max_ [mg/g]	
	0.979	19	8.8 × 10^−4^	112		0.969	190	0.002	377	
Redlich–Peterson	R^2^	χ^2^/DoF	K_RP_[dm^3^/mg]	a_RP_ ([dm^3^/mg])^B^	B	R^2^	χ^2^/DoF	K_RP_ [dm^3^/mg]	a_RP_ ([dm^3^/mg])^B^	B
	0.990	74.4	0.68	4.42 × 10^−9^	0.09	0.996	4.3	0.08	1.58 × 10^−7^	0.87

**Table 8:** The maximum adsorption capacity of various cheap adsorbents for copper adsorption.

Adsorbent	Capacity, q_max_	References
Dolomite	308–378 mg/g	[This study]
Bottom ash of expired drugs incineration	13.33 mg/g	[10]
Clay rocks	15 mg/g	[43]
Bottom ash of municipal waste incineration	24 mg/g	[44]
Algal biochar	104.16–227.27 mg/g	[4]
Dragon fruit peel	92.593 mg/g	[45]
Rambutan peel	192.308 mg/g	
Passion fruit peel	121.951 mg/g	
Grapefruit peel	80.6 mg/g	[46]
Orange peel	163 mg/g	[47]
Raw eggshell	94.59 mg/g	[48]
Chitosan	103 mg/g	[49]
Carbonized sunflower stem	38.05 mg/g	[50]
Fly ash	53.5 mg/g	[51]
